# Maternal depression and childhood injury risk: A population‐based cohort study in Denmark

**DOI:** 10.1002/brb3.2029

**Published:** 2021-01-15

**Authors:** Bente Kjær Lyngsøe, Trine Munk‐Olsen, Claus Høstrup Vestergaard, Dorte Rytter, Kaj Sparle Christensen, Bodil Hammer Bech

**Affiliations:** ^1^ Research Unit for General Practice Aarhus Denmark; ^2^ Department of Public Health Aarhus University Aarhus Denmark; ^3^ Aarhus University Hospital Aarhus Denmark; ^4^ National Centre for Register‐based Research Aarhus University Aarhus Denmark; ^5^ Lundbeck Foundation Initiative for Integrative Psychiatric Research (iPSYCH) Denmark

**Keywords:** Adolescent, Child, Delivery of Health Care, Depressive Disorder, Mother–Child Relations, Wounds and Injuries

## Abstract

**Aims:**

To assess the association between different stages of maternal depression and injury risk in offspring aged 0–10 years.

**Methods:**

Population‐based cohort study of all live‐born children in Denmark from 1 January 1997 until 31 December 2013 (*n* = 1,064,387). Main outcome measure was emergency department contacts with a main diagnosis of injury coded as DS00‐DT98 (chapter XIX) according to the ICD‐10. All information was obtained from Danish national registries.

**Results:**

Maternal depression was associated with higher injury hazard in the offspring throughout childhood compared to offspring of mothers with no history of depression. The strongest association was seen for the first year of life. *First‐time* maternal depression was most strongly associated with injury in the child, especially in the first year of life (aHR = 1.70, 95% CI: 1.48–1.96). Children of mothers with *relapse* depression had 1.57 higher hazard of injury in the first year of life (aHR: 1.57, 95% CI: 1.44–1.70). Children of mothers with previously treated depression (*post*depression) had 1.13 higher hazard of injury in the first year of life (aHR: 1.13, 95% CI: 1.09–1.17). Continuous treatment for depression was associated with a nonsignificant higher hazard of injuries in the first year of life (aHR: 1.06, 95% CI: 0.91–1.23).

**Conclusions:**

Maternal depression was associated with higher injury risk in the offspring, particularly in early childhood. The association persisted in children of mothers with relapse depression. Our results suggest that children of mothers with depression are vulnerable several years after depression onset and treatment cessation.

## Graphical abstract

This study adds to the field results from an entire nation on the association between maternal depression and offspring injury risk. Follow‐up is, to the best of our knowledge, one of the longest, and we show significant and interesting results.

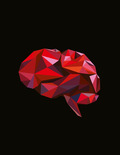

## 1 SIGNIFICANT OUTCOMES

- Children of mothers with depression have a higher risk of injuries both in the early years of treatment but also years after cessation of treatment.

## 2 LIMITATIONS

- In this register‐based study, it was not possible to identify nonpharmacologically treated mothers. They would be placed in the reference group and might attenuate an association.

## 3 INTRODUCTION

Injuries are a leading cause of death in children and adolescents (Nagaraja et al., 2005; Runyan et al., 2005). The most frequent types of injuries in the United States are fires, submersion/suffocation, falls, and poisoning (Nagaraja et al., 2005). Risk factors associated with childhood injury include parenting behavior (Schwebel & Brezausek, 2010), child health (Schwebel & Brezausek, 2009), child cognitive development (Schwebel et al., 2015), use of safety practices (Swartz et al., 2013), poverty, family size, illiteracy, single parenting, lack of control over environment, and mental health problems (Peden et al., 2008).

Depression is one of the most common mental disorders and a leading cause of disability (Murray & Lopez, 2013). It is a major public health concern worldwide, and one of many associated economic costs is adverse impact on the offspring (Bauer et al., 2016). Women have twice the risk of men to develop depression. During pregnancy and after birth, this risk increases even further (Angst et al., 2002; Munk‐Olsen et al., 2006). Several previous studies have found that maternal depression during childhood is associated with increased injury risk in the offspring (Baker et al., 2017; Phelan et al., 2007; Schwebel & Brezausek, 2008). Suggested mechanisms include reduced parental ability to maintain supervisory behavior (Baker et al., 2017; Phelan et al., 2007; Schwebel & Brezausek, 2008), altered risk‐taking behavior in the children (Phelan et al., 2014), behavioral problems in the children (Netsi et al., 2018), and reduced use of safety practices (McLennan & Kotelchuck, 2000). However, most previous studies rely on self‐reported information on injury, which can be prone to bias. Furthermore, several studies are based on active current depression (Phelan et al., 2007; Schwebel & Brezausek, 2008). One study used a large primary care database to explore the risk of childhood injuries in children exposed to maternal depression or anxiety (Baker et al., 2017), this study found episodes of maternal depression or anxiety to be associated with increased rates of child poisoning, fractures, and burns in the first five years of life.

### 3.1 Aims of the study

The aim of this large‐scale population‐based study was to examine the association between different stages of maternal depression and injury risk in the offspring using information from national health registries.

## 4 METHODS

### 4.1 Design

This large‐scale population‐based cohort study used national databases to follow all live‐born children in Denmark from birth and up to ten years of age. Data on the included children, their mothers, and all covariates were linked through the personal identification number assigned to all residents in Denmark at birth or immigration. The data were handled through Statistics Denmark in compliance with existing laws on data protection.

### 4.2 Participants

We included all children with Danish residency who were born in the period from 1 January 1997 until 31 December 2013 (*n* = 1,064,387) and their mothers (*n* = 618,681). The children were identified in the Danish National Patient Register and linked to their mother through the personal identification number in the Danish Civil Registration System (Caspar Thygesen & Ersbøll, 2011; Lynge et al., 2011; Pedersen, 2011). Children who died, disappeared, or emigrated during follow‐up were excluded from the analyses on the date of loss to follow‐up (*n* = 20,127). For each included child, follow‐up lasted until the 10th birthday, exclusion, or end of follow‐up (31 December 2013), whichever came first.

### 4.3 Main outcome

The outcome of interest was injuries in the offspring. This information was obtained from the Danish National Patient Register (Caspar Thygesen & Ersbøll, 2011; Lynge et al., 2011). An injury was defined as an emergency department contact with a main diagnosis coded as DS00‐DT98 in Chapter XIX of the International Classification of Diseases, 10th revision (ICD‐10), that is, injury, poisoning, and certain other consequences of external causes.

### 4.4 Exposure

Maternal depression was defined from inpatient or outpatient hospital admissions with a main diagnosis of depression according to the ICD‐10 and redemption of prescribed antidepressant medication Table 1. Certain antidepressants primarily used to treat other conditions such as chronic pain conditions and smoking cessation was excluded (N06AA and N06AX). Duration of depression treatment was calculated from dosage and packet size of prescriptions and predefined time intervals (three months following an outpatient contact and six months following admittance to a hospital).

**TABLE 1:** Criteria used to define depression events

Exposure measures	Specifications	Criteria for depression events
Antidepressant medications	N06AB03, N06AB04, N06AB05, N06AB06, N06AB08, N06AB10, N06AF01, N06AG02, N06AX03, N06AX06, N06AX11, N06AX16, N06AX21, N06AX22	The second redeemed prescription within 2 months was considered an event. The woman was assumed depressed for a period calculated from dosage and size of the prescribed packet.
Outpatient contact to a hospital	Main diagnosis of depression according to ICD−10: F32.0, F32.1, F32.2, F32.3, F32.8, F32.9, F33.0, F33.1, F33.2, F33.3, F33.4, F33.8, F33.9	The second outpatient contact within 2 months was considered an event. The woman was assumed depressed for the following 90 days.
Admission to a hospital	Main diagnosis of depression according to the ICD−10: F32.0, F32.1, F32.2, F32.3, F32.8, F32.9, F33.0, F33.1, F33.2, F33.3, F33.4, F33.8, F33.9	Inpatient admission to the hospital was considered an event. The woman was assumed depressed for the following 180 days.

Existing literature has shown that the duration or timing of depression influence associations with a range of outcomes. One study investigated “ever” depression and “persistent” depression and found persistent depression more strongly associated with the use of corporal punishment, infant hospitalization, and lower use of smoke alarms (Chung et al., 2004). Another study examined healthcare use in children of mothers treated with antidepressant medication. They defined treatment groups as nonusers, continuous users, starters, irregular users, and stoppers. They found differences in outcome for these groups. To the best of our knowledge, previous research has not explored the timing of maternal depression and offspring injury. Since the timing seems important in other areas, a priori groupings were made in this study to investigate the association between timing of depression and child injury risk. Maternal depression was divided into five categories of time‐dependent variables, which allowed change of category during follow‐up. A *depression event* was defined as fulfillment of any of the criteria described in Table 1.

- **No depression** (reference group). When a woman had not (yet) any records of a depression event, she was categorized with no depression.
- **First‐time depression**: When a woman had a record of a depression event for the first time, she was categorized with first‐time depression. The duration of first‐time treatment was set to a maximum of one year.
- **Continuous depression**: When a woman had several events after the first‐time depression period, she was categorized with continuous depression.
- **Postdepression**: Once the depression events stopped occurring, she was categorized with postdepression.
- **Relapse depression**: If a woman from the postdepression group had further events, she was categorized with relapse of depression.

Information on redemption of antidepressant medication was obtained from the Danish Register of Medicinal Product Statistics (Kildemoes et al., 2011), and information on (inpatient and outpatient) contacts was acquired from the Danish Psychiatric Central Research Register (Mors et al., 2011). Information on reimbursement of antidepressant medication and on hospital admittance was available from 1995 onwards.

### 4.5 Covariates

A priori confounders considered in this study included socioeconomic factors, maternal factors, paternal factors, and child factors (sex, gestational age, and age). Maternal age at birth (continuous) and current age (continuous), sex of the child, and calendar year were obtained from the Danish Civil Registration System (Caspar Thygesen & Ersbøll, 2011). Parity (1, 2, 3, 4, or more) and gestational age (birth before 37 completed gestational weeks (yes/no)) were obtained from the Danish National Patient Register (Lynge et al., 2011). These covariates were included as covariates recorded at the time of birth. Maternal income (low, moderate, high), paternal income (low, moderate, high), maternal educational level (≤10 years, 10–15 years, >15 years), and maternal cohabitation status (married, widowed, divorced, unmarried) were obtained from Statistics Denmark.(24) Paternal depression status (first time, continuous, relapse, and post) was obtained from the Danish Psychiatric Central Research Register (Mors et al., 2011), and the Danish Register of Medicinal Product Statistics (Kildemoes et al., 2011). Since other parental diseases have been found to be associated with higher offspring injuries (Chen et al., 2015), a variety of comorbidities was included as covariates.

Maternal and paternal comorbidities (see Appendix, Table A1) were identified by the algorithm developed by Prior et al (Prior et al., 2016). Each comorbid condition was included as a binary variable based on information from the Danish Psychiatric Central Research Register, the Danish National Patient Register, and the Danish Register of Medicinal Product Statistics (Kildemoes et al., 2011; Lynge et al., 2011; Mors et al., 2011). All comorbid conditions were included as time‐dependent covariates to allow for change over time.

### 4.6 Statistical analysis

All analyses were performed using Stata 15.0. We used multiple‐failure Cox regression as this model allowed us to investigate multiple injuries in each child rather than only the time to first injury. Cluster robust variance estimation was applied at maternal level to account for dependence of within‐child and within‐mother measurements (as the same mother may be included with multiple children). The proportional hazards assumption was tested for all covariates (Garret,; Westbury et al., 2016). Although some tests proved statistically significant, graphic inspection of the Schoenfeld residuals showed reasonable proportionality of covariates,the significance was most likely due to the massive dataset. Injury incidence rates were plotted by type of injury. Results from the Cox model are presented as adjusted hazard ratios (aHR) with 95% confidence intervals (CI) Table 3. Regressions were performed for each of the most prevalent injuries, and children of mothers with depression were compared to children of mothers with no depression. Missing data in any covariate were included as an *unknown* category in the analysis. Since the hazard ratio was not proportional with the age of the child, the risk was calculated in age groups in which the proportional hazards assumption seemed likely to hold. As boys had higher prevalence of injuries than girls did, a stratified analysis was performed by sex.

Multiple contacts originating from the same injury could have introduced bias. For example, removal of bandages or stitches could have resulted in two identical diagnosis codes in the Danish National Patient Register that were recorded weeks apart. To investigate this potential bias, we performed two subanalyses in which the adjusted regression models included a suspension period of 14 days and 49 days, respectively, in which no risk time was accrued, and no subsequent injuries with the exact same code were included.

Misclassification could arise from antidepressant medication used for treatment of other illnesses (Gardarsdottir et al., 2007). To address this potential bias, we conducted a subanalysis restricting antidepressant medication to prescriptions with an indication code specific to depression, which has been available since 2004. The sample for this analysis was restricted to children born from 1 January 2006 to 31 December 2013.

Depression prevalence increased during follow‐up. Therefore, we stratified results by birth before or after 2007 in a subanalysis.

Furthermore, we intended to perform a subanalysis of the risk of fatal accidents for children of mothers with depression compared to children of mothers without depression. However, we identified only 244 children who died from an accident as the immediate cause of death. Of these, 218 were unexposed to maternal depression, and 26 children were exposed (all categories combined, i.e., first time, continuous, relapse, and postdepression). No further analysis was made due to the low numbers.

### 4.7 Ethics approval

The study was approved by the Danish Data Protection Agency. According to Danish law, the study did not require approval from the Committee on Health Research Ethics of the Central Denmark Region as no biomedical intervention was performed.

## 5 RESULTS

### 5.1 Characteristics of the population

Compared to mothers with no records of depression, mothers treated for depression had several characteristics: They tended to have shorter education and lower income level, more were divorced or unmarried, and more had comorbidities. Compared to children of nondepressed mothers, children of exposed mothers were more often born preterm (<37 weeks of gestation), had more siblings (parity), and more often had a father with depression and comorbidities. More exposed children were born at the end of the study period Table 2.

**TABLE 2:** Characteristics of participants in all exposure groups (1,000 person‐years)

Depression	No (ref)	First time	Continuous	Post	Relapse	Total
1,000 person‐years	6,790.4 (85.4)	53.3 (0.7)	110.8 (1.4)	807.4 (10.2)	186.9 (2.4)	7,948.7 (100)
Age of mother	30.3 (4.7)	29.5 (5.0)	30.7 (4.9)	30.0 (5.3)	30.1 (5.1)	30.3 (4.8)
Year of birth						
1997–1999	1,889.1 (27.8)	12.2 (22.9)	18.9 (17.0)	151.0 (18.7)	39.5 (21.2)	2,110.8 (26.6)
2000–2003	1,790.8 (26.4)	14.1 (26.4)	29.1 (26.3)	201.2 (24.9)	50.6 (27.1)	2,085.8 (26.2)
2004–2006	1,488.0 (21.9)	12.6 (23.6)	30.2 (27.2)	201.4 (24.9)	47.1 (25.2)	1,779.3 (22.4)
2007–2009	1,009.8 (14.9)	9.5 (17.8)	21.2 (19.1)	152.4 (18.9)	32.7 (17.5)	1,225.6 (15.4)
2010–2013	612.6 (9.0)	5.0 (9.3)	11.4 (10.3)	101.4 (12.6)	16.9 (9.0)	747.3 (9.4)
Maternal education						
≤10 yrs	1,082.2 (15.9)	13.4 (25.1)	25.8 (23.3)	229.2 (28.4)	53.0 (28.3)	1,403.5 (17.7)
>10 & ≤15 yrs	3,187.8 (46.9)	24.8 (46.6)	50.6 (45.7)	357.9 (44.3)	83.7 (44.8)	3,704.8 (46.6)
>15 yrs	2,406.3 (35.4)	14.2 (26.7)	33.3 (30.1)	206.6 (25.6)	47.3 (25.3)	2,707.8 (34.1)
Unknown	114.1 (1.7)	0.9 (1.7)	1.0 (0.9)	13.7 (1.7)	2.9 (1.5)	132.6 (1.7)
Maternal income						
Low	402.8 (5.9)	2.6 (4.8)	4.4 (4.0)	39.5 (4.9)	8.1 (4.4)	457.4 (5.8)
Moderate	3,152.8 (46.4)	31.3 (58.7)	67.0 (60.4)	480.2 (59.5)	117.0 (62.6)	3,848.2 (48.4)
High	3,234.8 (47.6)	19.5 (36.5)	39.4 (35.5)	287.7 (35.6)	61.7 (33.0)	3,643.1 (45.8)
Civil status						
Widow	13.8 (0.2)	0.2 (0.3)	0.4 (0.3)	3.3 (0.4)	0.7 (0.4)	18.3 (0.2)
Divorced	369.6 (5.4)	4.5 (8.4)	12.7 (11.5)	96.0 (11.9)	22.6 (12.1)	505.3 (6.4)
Married	4,637.1 (68.3)	33.5 (62.8)	68.3 (61.6)	459.4 (56.9)	109.9 (58.8)	5,308.1 (66.8)
Unmarried	1,765.0 (26.0)	15.1 (28.4)	29.4 (26.6)	248.6 (30.8)	53.7 (28.7)	2,111.9 (26.6)
Unknown	4.8 (0.1)	0.0 (0.1)	0.0 (0.0)	0.2 (0.0)	0.0 (0.0)	5.1 (0.1)
Paternal depression						
No	6,264.3 (92.3)	45.8 (86.0)	89.7 (81.0)	659.4 (81.7)	149.4 (79.9)	7,208.6 (90.7)
Post	385.5 (5.7)	4.9 (9.2)	13.6 (12.2)	113.5 (14.1)	26.0 (13.9)	543.5 (6.8)
First time	23.9 (0.4)	0.6 (1.1)	0.9 (0.8)	4.7 (0.6)	1.6 (0.9)	31.7 (0.4)
Continuous	45.5 (0.7)	0.7 (1.4)	3.0 (2.7)	10.8 (1.3)	3.5 (1.9)	63.5 (0.8)
Relapse	71.2 (1.0)	1.3 (2.4)	3.6 (3.2)	19.1 (2.4)	6.3 (3.4)	101.5 (1.3)
Maternal mental comorbidities						
No	5,778.4 (85.1)	40.1 (75.3)	71.7 (64.7)	588.2 (72.8)	125.9 (67.4)	6,604.2 (83.1)
Yes	1,012.0 (14.9)	13.2 (24.7)	39.1 (35.3)	219.2 (27.2)	61.0 (32.6)	1,344.5 (16.9)
Paternal mental comorbidities						
No	5,856.7 (86.3)	44.4 (83.3)	88.6 (79.9)	659.1 (81.6)	150.6 (80.6)	6,799.3 (85.5)
Yes	933.7 (13.7)	8.9 (16.7)	22.2 (20.1)	148.4 (18.4)	36.3 (19.4)	1,149.4 (14.5)
Parity						
1	2,996.8 (44.1)	22.8 (42.8)	45.7 (41.2)	337.3 (41.8)	74.9 (40.1)	3,477.5 (43.7)
2	2,566.1 (37.8)	20.1 (37.6)	42.8 (38.7)	285.2 (35.3)	69.7 (37.3)	2,983.9 (37.5)
3	929.0 (13.7)	7.5 (14.1)	16.2 (14.7)	124.9 (15.5)	28.9 (15.5)	1,106.6 (13.9)
>4	298.5 (4.4)	2.9 (5.5)	6.0 (5.4)	60.0 (7.4)	13.3 (7.1)	380.8 (4.8)
Preterm birth (<37 weeks of gestational age)						
Yes	404.0 (5.9)	3.6 (6.8)	8.7 (7.8)	58.7 (7.3)	13.9 (7.4)	488.9 (6.2)
No	6,369.4 (93.8)	49.6 (93.0)	101.8 (91.9)	746.8 (92.5)	172.6 (92.4)	7,440.2 (93.6)
Unknown	17.0 (0.3)	0.1 (0.2)	0.3 (0.2)	1.9 (0.2)	0.4 (0.2)	19.6 (0.2)
Child gender						
Boy	3,480.6 (51.3)	27.3 (51.2)	56.7 (51.1)	416.4 (51.6)	95.7 (51.2)	4,076.7 (51.3)
Girl	3,309.7 (48.7)	26.0 (48.8)	54.1 (48.9)	391.0 (48.4)	91.2 (48.8)	3,872.0 (48.7)

### 5.2 Descriptive results

During follow‐up, we recorded 1,228,550 injuries occurring in 536,474 children. The types of injuries were 712,157 extremity injuries (arms and legs), 380,804 head injuries, 28,622 neck/torso injuries, 37,863 foreign objects entering natural orifice (objects), 47,016 burns/corrosions and poisonings (burns), and 22,088 injuries to unspecified bodily region.

**FIGURE 1:**
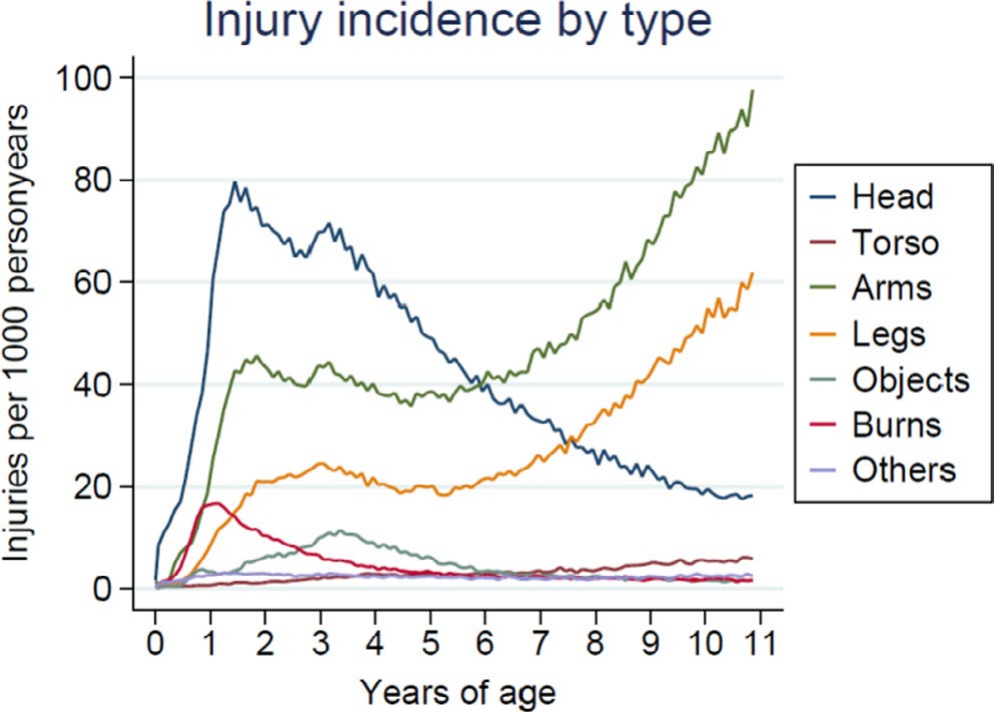
Plots of types of injuries per 1,000 person‐years stratified by age of children. In descending order are injuries to the head, neck/abdomen/thorax/pelvis (torso), upper extremity and shoulder (arm), lower extremity and hip (leg), foreign objects entering a natural orificium, burns/corrosions/ frostbites/poisonings. Others comprise unspecified/multiple/unspecific regions and other external causes of injuries

The incidence of head injuries, burns, and poisonings peaked in early childhood (1–3 years of age) Figure 1. Likewise, injuries to the arms and legs peaked in early childhood (1–3 years of age), and in later childhood (above 5 years of age) (Data not shown). Compared to girls, boys had higher incidence of head injuries, burns, and poisonings (Data not shown).

### 5.3 Main results

Maternal depression was associated with higher risk of injury in childhood across all age groups and exposure groups Tables 3 and 4. First‐time maternal depression was associated with the highest hazard throughout childhood, followed by relapse, post, and continuous depression. The association was strongest during the child's first year of life, and the main results from this period will be described in the following.

**TABLE 3:** Adjusted hazard ratio (95% CI) for any injury comparing children of mothers with depression to children of mothers with no depression, stratified by age (years)

	Age 0 years	Age 1 year	Age 2–4 years	Age 5–7 years	Age 8–10 years
Number of injuries	41,797	143,996	376,673	231,268	217,965
No depression*	1	1	1	1	1
First time	**1.70 (1.48 1.96)**	**1.17 (1.10 1.24)**	**1.12 (1.08 1.16)**	**1.12 (1.07 1.17)**	**1.12 (1.06 1.17)**
Continuous	1.06 (0.91 1.23)	**1.08 (1.01 1.15)**	**1.05 (1.01 1.08)**	1.00 (0.98 1.04)	**1.08 (1.05 1.11)**
Post	**1.13 (1.09 1.17)**	**1.11 (1.09 1.13)**	**1.09 (1.08 1.10)**	**1.07 (1.06 1.08)**	**1.07 (1.06 1.09)**
Relapse	**1.57 (1.45 1.70)**	**1.11 (1.07 1.16)**	**1.10 (1.08 1.12)**	**1.07 (1.04 1.09)**	**1.10 (1.07 1.12)**

Note

All estimates are adjusted for calendar year, child sex, gestational age (birth before 37 completed gestational weeks (yes/No)), child age, maternal age current and at birth (continuous), parity (1, 2, 3, 4, or more), maternal and paternal income (low, moderate, high), maternal educational level (≤10 years, 10–15 years, >15 years), maternal cohabitation status (married, widowed, divorced, unmarried), paternal depression status, and maternal and paternal comorbidities described further in appendix.

* Reference group was children of mothers without depression.

**TABLE 4:** Adjusted hazard ratio (95% CI) for any injury comparing children of mothers with depression to children of mothers with no depression, stratified by age and sex of the child

Exposure	Sex	Age 0 years	Age 1 year	Age 2–4 years	Age 5–7 years	Age 8–10 years
First time	Boy	1.78 (1.46 2.16)	1.16 (1.07 1.25)	1.10 (1.05 1.15)	**1.06 (1.00 1.13)**	**1.06 (0.99 1.13)**
	Girl	1.62 (1.33 1.97)	1.19 (1.08 1.30)	1.15 (1.09 1.22)	**1.21 (1.13 1.29)**	**1.18(1.10 1.26)**
Continuous	Boy	1.03 (0.84 1.26)	1.06 (0.98 1.15)	1.07 (1.02 1.11)	1.00 (0.96 1.03)	**1.03 (0.99 1.08)**
	Girl	1.10 (0.88 1.37)	1.10 (1.00 1.21)	1.02 (0.97 1.07)	1.03 (0.98 1.08)	**1.14 (1.09 1.19)**
Post	Boy	1.12 (1.07 1.18)	1.12 (1.09 1.15)	1.09 (1.07 1.10)	**1.05 (1.03 1.07)**	**1.06 (1.04 1.08)**
	Girl	1.14 (1.08 1.20)	1.10 (1.07 1.13)	1.09 (1.08 1.11)	**1.10 (1.08 1.12)**	**1.10 (1.08 1.12)**
Relapse	Boy	1.52 (1.36 1.70)	1.13 (1.07 1.19)	1.09 (1.06 1.12)	**1.04 (1.01 1.07)**	**1.07 (1.03 1.10)**
	Girl	1.63 (1.45 1.85)	1.09 (1.02 1.16)	1.12 (1.08 1.16)	**1.12 (1.08 1.16)**	**1.14 (1.10 1.18)**

Note

Effect modification by gender is tested using the Wald test. Estimates in bold indicate statistically significant difference. Reference group was children of mothers without depression.

All estimates are adjusted for calendar year, child sex, gestational age (birth before 37 completed gestational weeks (yes/No)), child age, maternal age current and at birth (continuous), parity (1, 2, 3, 4, or more), maternal and paternal income (low, moderate, high), maternal educational level (≤10 years, 10–15 years, >15 years), maternal cohabitation status (married, widowed, divorced, unmarried), paternal depression status, and maternal and paternal comorbidities described further in appendix.

*First‐time maternal depression* was associated with a 1.70 higher hazard of any injury in the child compared to no maternal depression (aHR: 1.70, 95% CI: 1.48–1.96). *Continuous* maternal depression was also associated with a higher hazard, albeit showing the smallest association in all exposure groups (aHR: 1.06, 95% CI: 0.91–1.23). *Relapse* maternal depression was associated with a 1.57 higher hazard of injury in the first year of life (aHR: 1.57, 95% CI: 1.44–1.70). *Postdepression* maternal depression was associated with a 1.13 higher hazard of injury in the first year of life (aHR: 1.13, 95% CI: 1.09–1.17) Table 3. From one year of age onwards, children of mothers in all depression groups had higher hazard of injury, although the hazard ratios were lower than during the first year of life. Children of mothers with depression had higher hazard ratios of all studied types of injury compared to children of mothers with no record of depression (data not shown). We found indications that the sex of the child modified the association between maternal depression and child injuries. At 5–7 years of age, girls had statistically significantly higher hazard ratios of any injury than boys in all exposure groups, except for continuous depression. At 8–10 years of age, girls had significantly higher hazard ratios than boys in all exposure groups Table 4.

### 5.4 Subanalyses

The subanalysis, which was designed to investigate potential bias from errors of counting the same injury twice or more, did not change our results substantially (see Appendix , Tables A2 and A3). Moreover, the use of the specific indication for depression on prescriptions did not alter the results substantially (see Appendix , TableA2). Stratification on birth year before and after 2007 showed a nonsignificantly higher hazard for children born after 2007 in all exposure groups (see Appendix, Table A2).

## 6 DISCUSSION

### 6.1 Summary of findings

Children of mothers with depression had a significantly higher risk of childhood injuries, even years after treatment cessation, compared to children of nondepressed mothers. This relative risk was highest in early childhood, but it remained statistically significant throughout childhood.

### 6.2 Strengths and limitations

A strength of the present study is the population‐based design, including comprehensive data on all children born in 1997–2013 and very little loss to follow‐up. The Danish registries are considered valid sources of information with few missing values (Kildemoes et al., 2011; Lynge et al., 2011; Mors et al., 2011; Pedersen, 2011), and they allow adjustment for various potential confounders. This limits the risk of biased results.

A limitation of the study is the identification of mothers with depression. The register‐based design is vulnerable to misclassification of nonpharmacologically treated depression and undiagnosed episodes of depression. In the present study, the prevalence of maternal depression was lower than described in the literature (Baker et al., 2017; Phelan et al., 2007). We did not have information about depressed mothers who did not receive neither medical treatment nor hospital diagnosis. They would be misclassified as healthy mothers. This potential misclassification might have caused an underestimation of the associations. Furthermore, antidepressants are used for other illnesses than depression. We addressed this potential bias by performing a subanalysis including only medication pertaining to an indication‐specific code for depression,this code has been available since 2004 (analysis performed on children born in 2006–2013). This did not alter our results significantly. An explanation could be that antidepressants are most often prescribed for depression (Gardarsdottir et al., 2007). or that depression accounts for the majority of the association in the main analysis. The exposure group might be viewed as heterogenous since it included both mothers receiving medication and mothers admitted to the hospital or seen in an outpatient clinic. Nearly all women from the hospital/outpatient clinic setting also received medication, and the group with admissions or outpatient contacts was much smaller. A plausible speculation might be that the mothers admitted or seeing a psychiatrist in outpatient clinics would be more severely affected than the peers only receiving medication. However, subgroup analyses (data not shown) showed no difference in association with childhood injuries for children of mothers with mild depression (antidepressant medication only) and children of mothers with severe depression (admissions and/or outpatient contacts).

Lastly, other potentially influential factors for which we had no available information, for example, social support and behavioral problems in the offspring, could also have been relevant to consider since a good network may alleviate negative consequences of maternal depression (Kingsbury et al., 2015; Skärsäter, 2006).

### 6.3 Generalizability

This population‐based study included all children live‐born in Denmark in the inclusion period. Thus, the findings reported in this study are generalizable to comparable nations with similar healthcare systems and similar populations.

## 7 COMPARISON WITH EXISTING LITERATURE

Previous studies have also found that maternal depression is associated with increased use of health care in the offspring, including more emergency department visits (Farr et al., 2013; Minkovitz et al., 2005; Sills et al., 2007). One study found a twofold higher risk of injury until six years of age for children of mothers with severe and persistent depressive symptoms (Phelan et al., 2007). An interesting aspect in this study was that children of mothers with depression had a higher risk of externalizing behavior, but this trait did not mediate the effect of depressive symptoms. These findings have been replicated and have shown severe maternal depression to be associated with higher risk of injury in the offspring until three years of age, whereas moderate depression was not related to higher childhood injury risk in the multivariate analyses (Schwebel & Brezausek, 2008). A proposed explanation was that less severely affected women might have enough energy to safeguard the home environment. Research on the use of safety practices, such as car seats, fire alarms, or safe storage of poisonous products at home, has reported conflicting results. One study found lower odds of using safety practices in mothers with depression (McLearn et al., 2006), which was supported by a review (Field, 2010). Another study found that depression was not independently associated with the use of safety practices, but lack of social support and stress were instead reported to explain the association (Mulvaney & Kendrick, 2006).

Our results are in line with the findings in a recent large‐scale study, which reported higher childhood injury rates during episodes of maternal depression (Baker et al., 2017). This study also included women with depressive symptoms without pharmacological treatment, which was reflected in a much higher prevalence of depression (26.4% had an episode of depression/anxiety during follow‐up). We add to existing research the findings that sex modified the association between maternal depression and child injury risk in late childhood, suggesting that maternal depression has a greater impact on injury risk in girls than boys in the age 5–10 years. This adds to existing literature showing increasing odds of injury with male sex (Nagaraja et al., 2005; Orton et al., 2012), and higher odds of injury in the age 1–2 years compared to the first year of age (Orton et al., 2012).

### 7.1 Implications

Our findings highlight that children of mothers with depression have a higher risk of childhood injuries compared to children of mothers with no record of depression. The finding that continuous depression had the lowest hazard, albeit not significantly across all age groups, might indicate that continued treatment alleviate part of the injury risk in the offspring. Parental and environmental strategies, such as increased supervision, parental modification of own risk reducing behavior, hazard removal, and safe storage of dangerous products, have been shown to reduce the risk of at‐home injuries, whereas teaching rules or prohibitions to promote safety can elevate injury risk in toddlers (Morrongiello et al., 2004). A recent study found that the vast majority of GPs find it relevant to talk to children of depressed mothers during consultation, but approximately 40% of the GPs assess their knowledge on the topic as insufficient and call for education and referral opportunities (Hansen et al., 2018). Barriers for engaging in prevention of childhood injury in general practice include lack of personal experience, limited resources, low self‐confidence, and lack of time (Woods, 2006). Parenting interventions have been found to be feasible and effective in reducing childhood injury, also in children of mothers with mental illness, including depression (Kendrick et al., 2013). In Denmark, pregnancy care programs and childcare programs offered in general practice constitute an opportunity for the GP to identify these vulnerable families. However, children of mothers with depression have higher risk of missing these routine childcare visits (Lyngsøe et al., 2018). Hence, for these suggestions to succeed, political incitement is needed to address the barriers described (Woods, 2006).

### 7.2 Conclusions

Children of mothers with current or past depression have a higher risk of childhood injury. The association was most pronounced for children of mothers with first‐time depression, but we also found higher risk for children of mothers with continuous, past, or relapse depression. The association was highest for children below one year of age. However, an association was found for all age groups until 10 years of age. The association persisted after adjustment for various potential confounders that could influence the injury risk.

## CONTRIBUTORS

BHB and BKL conceived the research idea. BKL wrote the first draft. CV, BHB, and BK contributed to data analysis, and BKL and CV analyzed all data. All authors participated in the interpretation of the results, revision of the paper, and approved of the final manuscript.

## PROVENANCE AND PEER REVIEW

Not commissioned, externally peer reviewed.

## TRANSPARENCY STATEMENT

The lead author (BKL) affirms that the manuscript is an honest, accurate, and transparent account of the study being reported; that no important aspects of the study have been omitted; and that any discrepancies from the study as planned have been explained.

## PATIENT AND PUBLIC INVOLVEMENT

It was not possible to involve patient or public in the design, or conduct, or reporting, or dissemination of this study.

## COMPETING INTEREST

None.

## **AUTHOR** **CONTRIBUTIONS**

Bodil Hammer Bech and Bente Kjær Lyngsøe outlined the idea of the study. Claus Høstrup Vestergaard and Bente Kjær Lyngsøe performed the statistical analyses. Bodil Hammer Bech contributed to supervision. All authors contributed equally to planning the study and interpreting the results, and have contributed to alterations and revisions of all drafts of the manuscript.

### Peer Review

The peer review history for this article is available at https://publons.com/publon/10.1002/brb3.2029.

## Supporting information

Supplementary Material

## Data Availability

The data that support the findings of this study are stored and maintained electronically at Statistics Denmark and are not publicly available.
