# Supplementary material for: Maternal depression and childhood injury risk: A population‐based cohort study in Denmark
Source: Brain Behav. 2021 Jan 15;11(3):e02029. doi: 10.1002/brb3.2029 (PMC7994683; doi:10.1002/brb3.2029)
Supplement: Supplementary file 1 — Supplementary Material [file BRB3-11-e02029-s001.docx]

**Appendix**

**Table A1: Comorbidities included in the adjusted analyses as binary variables (yes/no)**

| **Category** | **Disease group** |
| --- | --- |
| **Circulatory system**  **Endocrine system**  **Pulmonary system and**  **allergy**  **Gastrointestinal system**  **Urogenital system**  **Musculoskeletal system**  **Hematological system**  **Cancers**  **Neurological system**  **Mental health conditions** | Hypertension  Dyslipidemia  Ischemic heart disease  Atrial fibrillation  Heart failure  Peripheral artery occlusive disease  Stroke  Diabetes mellitus, types 1 and 2  Thyroid disorder  Gout  Chronic pulmonary disease  Allergy  Ulcer/chronic gastritis  Chronic liver disease  Inflammatory bowel disease  Diverticular disease of intestine  Chronic kidney disease  Connective tissue disorder  Osteoporosis  Painful condition  Anemia  HIV/AIDS  Cancer  Vision problem  Hearing problem  Migraine  Epilepsy  Parkinson disease  Multiple sclerosis  Neuropathy  Psychological distress  Alcohol problem  Substance abuse  Anorexia/bulimia  Bipolar affective disorder  Schizophrenia or schizoaffective disorder Dementia |
| Abbreviations: AIDS, acquired immunodeficiency syndrome; HIV, human immunodeficiency virus | |
| Reference ^22^ | |

**Sub-analyses**

| **Table A2: Subanalyses including ordination-specific code and year** | | | | |
| --- | --- | --- | --- | --- |
| **Exposure** | **Main cox^1^** | **Indo cox^2^** | **<2007^3^** | **≥2007^3^** |
| No depression | Ref. | Ref. | Ref. | Ref. |
| First time | **1.14 (1.11 1.16)** | **1.12 (1.09 1.14)** | **1.12 (1.10 1.15)** | **1.20 (1.14 1.26)** |
| Continuous | **1.05 (1.03 1.07)** | 1.03 (1.01 1.06) | **1.05 (1.03 1.07)** | **1.04 (1.00 1.08)** |
| Relapse | **1.10 (1.08 1.11)** | **1.09 (1.07 1.11)** | **1.09 (1.07 1.11)** | **1.14 (1.11 1.17)** |
| Post | **1.08 (1.08 1.09)** | **1.07 (1.06 1.08)** | **1.08 (1.07 1.09)** | **1.10 (1.08 1.12)** |
| ^1^Results from the main analysis of this study  ^2^Subanalysis including only prescriptions for antidepressants with a code for depression. Study population restricted to children born from 1 January 2006 to 31 December 2013  ^3^Calendar year for child birth | | | | |

| **Table A3: subanalysis including quarantine period of 14 and 49 days.** | | | |
| --- | --- | --- | --- |
| **Exposure** | **Main analysis^1^** | **14 days of suspension^2^** | **49 days of suspension^3^** |
| No depression | Ref. | Ref. | Ref. |
| First time | 1.138 (1.113 1.165) | 1.131 (1.105 1.157) | 1.128 (1.102 1.154) |
| Continuous | 1.050 (1.031 1.070) | 1.051 (1.031 1.070) | 1.045 (1.026 1.065) |
| Relapse | 1.099 (1.084 1.113) | 1.099 (1.084 1.114) | 1.098 (1.083 1.113) |
| Post | 1.083 (1.075 1.092) | 1.086 (1.077 1.094) | 1.087 (1.079 1.095) |
| ^1^Results from the main analysis of this study  ^2^After first reported injury, a suspension period of 14 days followed during which no risk time was accrued, and any injuries with the same code were ignored.  ^3^After first reported injury, a suspension period of 49 days followed during which no risk time was accrued, and any injuries with the same code were ignored. | | | |
